# Complement C3 and marginal zone B cells promote IgG-mediated enhancement of RBC alloimmunization in mice

**DOI:** 10.1172/JCI167665

**Published:** 2024-04-15

**Authors:** Arijita Jash, Thomas Pridmore, James B. Collins, Ariel M. Hay, Krystalyn E. Hudson, Chance John Luckey, James C. Zimring

**Affiliations:** 1 University of Virginia School of Medicine, Charlottesville Virginia, USA.; 2 Carter Immunology Center, University of Virginia, Charlottesville, Virginia, USA.; 3 Department of Pathology and Cell Biology, Columbia University Irving Medical Center, New York, New York, USA.

**Keywords:** Immunology, Adaptive immunity

## Abstract

Administration of anti-RhD immunoglobulin (Ig) to decrease maternal alloimmunization (antibody-mediated immune suppression [AMIS]) was a landmark clinical development. However, IgG has potent immune-stimulatory effects in other settings (antibody-mediated immune enhancement [AMIE]). The dominant thinking has been that IgG causes AMIS for antigens on RBCs but AMIE for soluble antigens. However, we have recently reported that IgG against RBC antigens can cause either AMIS or AMIE as a function of an IgG subclass. Recent advances in mechanistic understanding have demonstrated that RBC alloimmunization requires the IFN-α/-β receptor (IFNAR) and is inhibited by the complement C3 protein. Here, we demonstrate the opposite for AMIE of an RBC alloantigen (IFNAR is not required and C3 enhances). RBC clearance, C3 deposition, and antigen modulation all preceded AMIE, and both CD4^+^ T cells and marginal zone B cells were required. We detected no significant increase in antigen-specific germinal center B cells, consistent with other studies of RBC alloimmunization that show extrafollicular-like responses. To the best of our knowledge, these findings provide the first evidence of an RBC alloimmunization pathway which is IFNAR independent and C3 dependent, thus further advancing our understanding of RBCs as an immunogen and AMIE as a phenomenon.

## Abstract 2

Complement C3 and marginal zone B cells are mechanistically involved in IgG2c-mediated enhancement of RBC alloimmunization but interferon is not required.

## Introduction

Immunoglobulins are most commonly thought of as immune effector molecules. However, it has been known for over a century that Igs can also substantially enhance primary humoral immunity to soluble or small particulate antigens (1–3), referred to as antibody-mediated immune enhancement (AMIE). Conversely, hemolytic disease of the fetus and newborn (HDFN) has been greatly decreased by suppression of maternal alloimmunization through the administration of polyclonal plasma–derived anti-RhD IgG (4, 5), referred to as antibody-mediated immune suppression (AMIS). Although the mechanisms of AMIS and AMIE remain controversial (6–9), the above findings have led to a proposed paradigm, that IgG enhances responses to antigens that are soluble or on small particulate matter like viruses, but IgG suppresses responses to large cellular antigens such as RBCs (7, 10, 11). Indeed, Enriquez-Rincon et al. showed that IgG against a single antigen causes enhancement if the antigen is soluble, but suppression if it is on a RBC (12). However, we have recently contributed a nuance to the paradigm, in that the IgG subclass regulates AMIE versus AMIS for the same antigen target on RBCs in a murine model of alloimmunization (13).

IgG2a, IgG2b, and IgG2c greatly enhance antibody responses to RBCs expressing the human Kell antigen, whereas IgG1 suppresses these responses. We observed no significant effect for IgG3. In one report, analogous findings were observed in human clinical studies that showed that the same anti-RhD antibody suppressed or enhanced humoral responses to infused RhD^+^ RBCs based upon the constant region of the antibody and the cells in which the antibody was expressed (14). Although it has been speculated that the enhancement is due to posttranslational modifications (15, 16), these findings nevertheless demonstrate that in humans, as in mice, antibodies against RBC antigens can cause AMIE, given the chemistry of the constant region. To the best of our knowledge, only IgG1 and IgG3 (not IgG2 and IgG4) have been tested in humans (17).

In recent years, it has become clear that the immunological properties of RBCs are distinct from other antigens, i.e., they require a spleen (18, 19), marginal zone (MZ) B cells (20, 21), and signaling through the IFN-α/-β receptor IFNAR (22–25), and they are suppressed by complement C3 (26, 27), often have rapid senescence of antibodies (28–30), and do not induce an appreciable antigen-specific primary germinal center (GC) response (20, 24, 31, 32). In this context, the current report is a mechanistic analysis of IgG2c-induced AMIE for an RBC antigen. Here, we report that IgG2c-induced AMIE was distinct from previously described mechanisms of RBC alloimmunization, in that it did not require IFNAR and C3 contributed to immunization. IgG2c-induced AMIE is also distinct, as it requires Fc γ receptors (FcgRs) that drive consumption of opsonized RBCs and/or antigens. Common to other circumstances that promote RBC alloimmunization, in AMIE both CD4^+^ T cells and MZ B cells were required, and GC responses were not detectable. Together, the findings in this study elucidate mechanistic properties of AMIE, with RBCs as an immunogen that are relevant to alloimmunization in transfusions, the maternal/fetal interface, and the emerging use of RBCs as a cellular therapy for immune modulation and immunization against pathogens and cancer (33–35).

## Results

### IgG2c causes antigen modulation without increased complement C3 deposition during AMIE.

**Figure 1:**
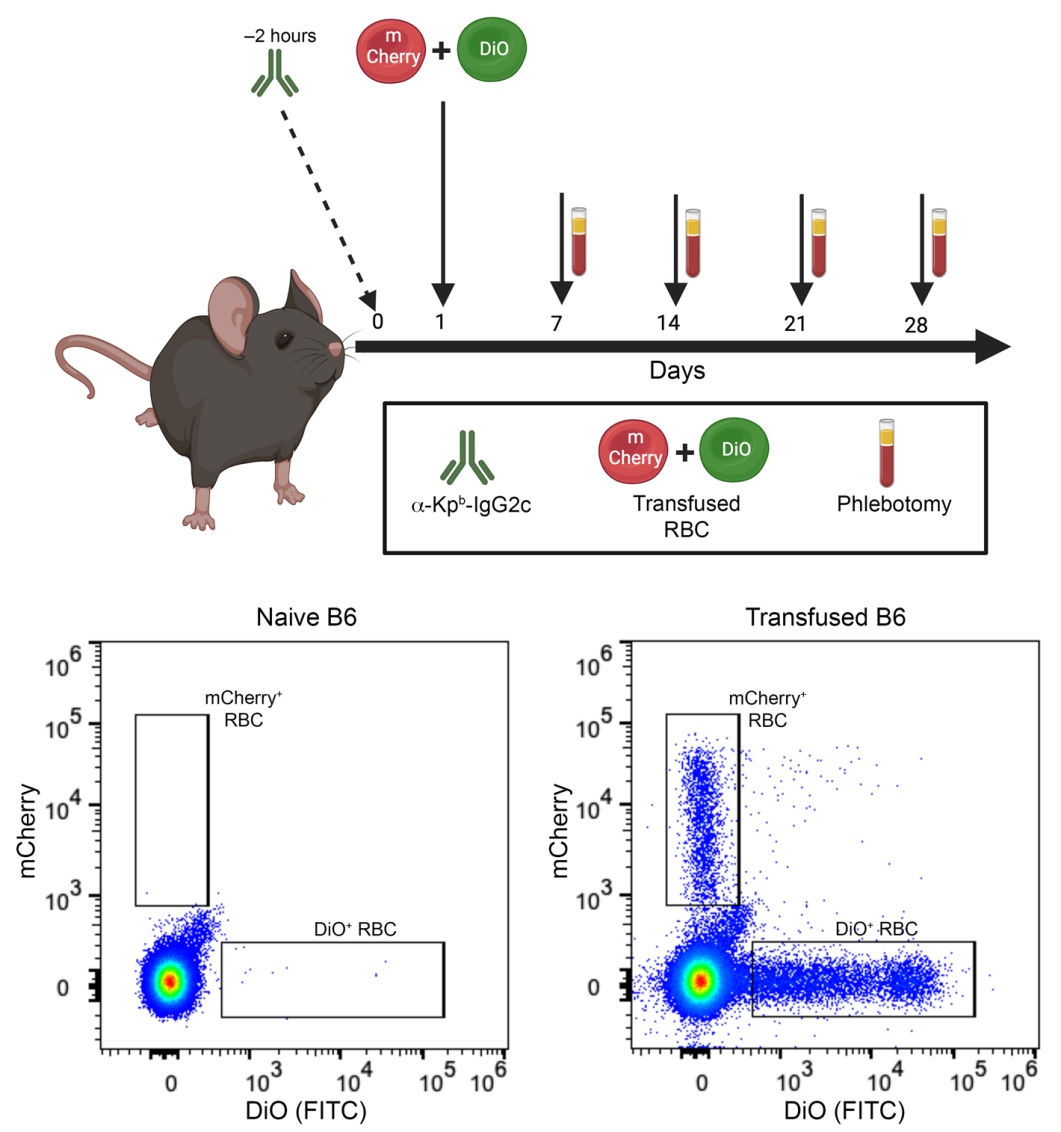
General experimental design. General schema of the experimental design used throughout this study. For IgG2c-induced AMIE, anti-Kp^b^ (α-Kp^b^) was infused 2 hours prior to transfusion. Transfusion consisted of DiO-labeled KEL-K2^lo^ RBCs mixed at a 1:1 ratio with transgenic mCherry–expressing RBCs. Blood samples were taken at the indicated time points. Serum was analyzed for anti-KEL IgM and anti-KEL IgG, while RBCs were monitored for post-transfusion survival, antibody binding to the RBC, antigen modulation, and C3 deposition (methods for each analysis are presented in subsequent sections; Figures 2–4, Figure 6, A, B, D, and E, and Figure 7, B, C, E, and F). Representative flow cytometric plots are shown to indicate the empty gates in untransfused (naive) mice and the visualized DiO^+^ and mCherry^+^ cell populations in transfusion recipients. This figure was prepared with BioRender.com.

The KEL-K2^lo^ mouse expresses low levels of the human Kell glycoprotein variant with epitopes of K2, Kp^b^, and Js^b^ (36). Monoclonal anti-Kp^b^ IgG2c was injected intravenously 2 hours before transfusion, followed by transfusion of a 1:1 mixture of DiOC18(3)-labeled (DiO-labeled) KEL-K2^lo^ RBCs and transgenic mCherry RBCs. The general experimental design, depicted in Figure 1, was utilized throughout the rest of the study, without alteration unless noted. Representative flow cytometric plots are shown to demonstrate post-transfusion enumeration of transfused RBCs (Figure 1).

**Figure 2:**
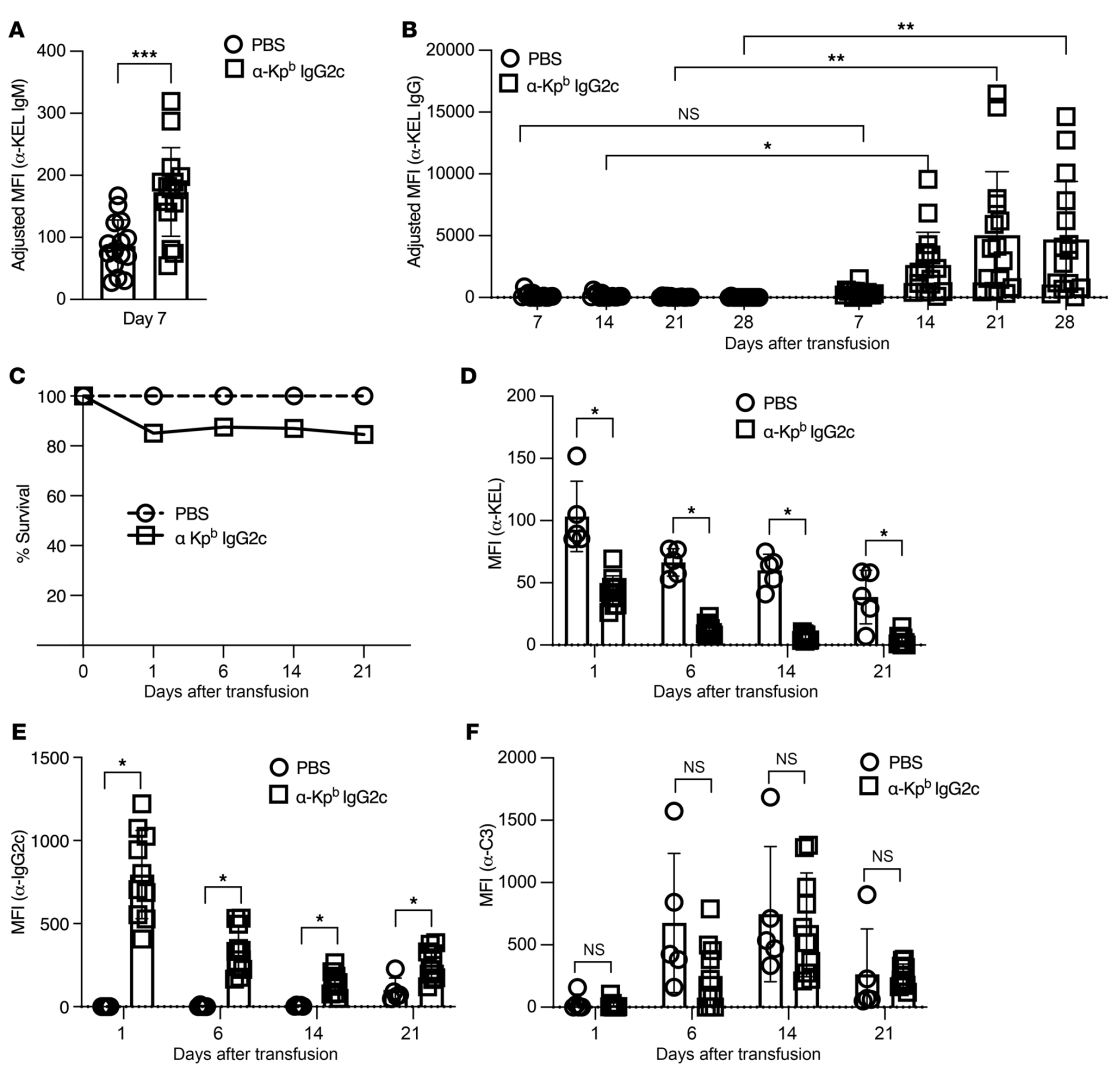
RBC clearance, antigen modulation and C3 deposition during IgG2c-induced AMIE. (**A** and **B**) Using the experimental design in Figure 1, anti-Kp^b^ IgG2c enhanced anti-KEL IgM at day 7 and IgG at day 14. (**C**) Anti-Kp^b^ IgG2c caused approximately 15% clearance of KEL-K2^lo^ RBCs by 24 hours, with no additional clearance at later time points. (**D**) RBCs were stained with anti-KEL, which is not blocked by prebound anti-Kp^b^, to assess antigen modulation over time, which had increased magnitude and kinetics in mice treated with anti-Kp^b^. (**E**) RBCs were stained with anti-IgG2c to test the amount of antibody coating RBCs. As predicted, RBCs were coated in mice that received anti-Kp^b^ IgG2c but not in PBS-treated control mice. (**F**) RBCs were stained with anti-C3 to assess the amount of C3 fixed to the RBC surface. All experiments were carried out a minimum of 3 times with 10–15 (**A** and **B**) and 6–10 (**C**–**F**) mice per group with similar results. *P* values were calculated using a multiple Mann-Whitney test *U* test and are designated as *P* > 0.05 (NS) and *P < 0.05, **P < 0.01, and ***P < 0.0005, by multiple Mann-Whitney *U* test (**A** and **C**–**F**) and repeated-measures, 2-way ANOVA with -Šidák’s multiple-comparisons test (**B**).

Consistent with our previous report (37), anti-Kp^b^ IgG2c enhanced both early production of anti–Kell IgM and later production of anti–Kell IgG (Figure 2, A and B). Also consistent with our previous report, KEL-K2^lo^ RBCs induced IgM but not IgG on its own (i.e., PBS control) (37). Approximately 15% of KEL-K2^lo^ (DiO^+^) RBCs cleared by 24 hours, after which no additional clearance was observed (Figure 2C). We measured antigen modulation by staining with a monoclonal antibody that recognizes an unidentified epitope on the KEL-K2 gene product (anti-KEL)) that is not blocked by anti-Kp^b^ (37). This avoided the problem of decreased staining due to antigen masking and allowed direct assessment of the amount of Kell glycoprotein on RBCs. The anti–Kp^b^ IgG2c induced 50% antigen modulation by 24 hours, with ongoing modulation to low residual levels by day 6 (Figure 2D). Coating of KEL-K2^lo^ RBCs with the injected anti-Kp^b^ (i.e., direct antiglobulin test [DAT]) was measured by staining with anti-IgG2c. As predicted, we detected a strong signal on KEL-K2^lo^ but not control RBCs at 24 hours (Figure 2E); the DAT decreased over the time course in parallel with decreased anti-KEL staining, suggesting antigen modulation of both epitopes. By day 21, the DAT was weakly positive on KEL-K2^lo^ RBCs in control mice pretreated with PBS (Figure 2E) and antigen modulation was observed (Figure 2D). We interpret this as low-level spontaneous immunization against KEL-K2^lo^ RBCs that then caused antigen modulation. The anti–KEL IgG was probably not detected in serum from PBS-treated mice at the same point (21 days) (Figure 2B), as the anti-KEL antibody was bound to circulating KEL-K2^lo^ RBCs.

Staining with anti-C3 antibody demonstrated deposition that was first detected by day 6 in both the anti–Kp^b^ IgG2c antibody and PBS-treated groups, with no significant differences noted (Figure 2F). RBC surface C3 remained elevated until day 14 and then decreased by day 21. Anti-C3 staining specifically recognized C3, as control incubations with opsonized RBCs showed strong signal in serum from WT but not C3-KO mice (Supplemental Figure 1; supplemental material available online with this article; https://doi.org/10.1172/JCI167665DS1).

### IFN signaling through the IFNAR is not required for IgG2c-induced AMIE.

**Figure 3:**
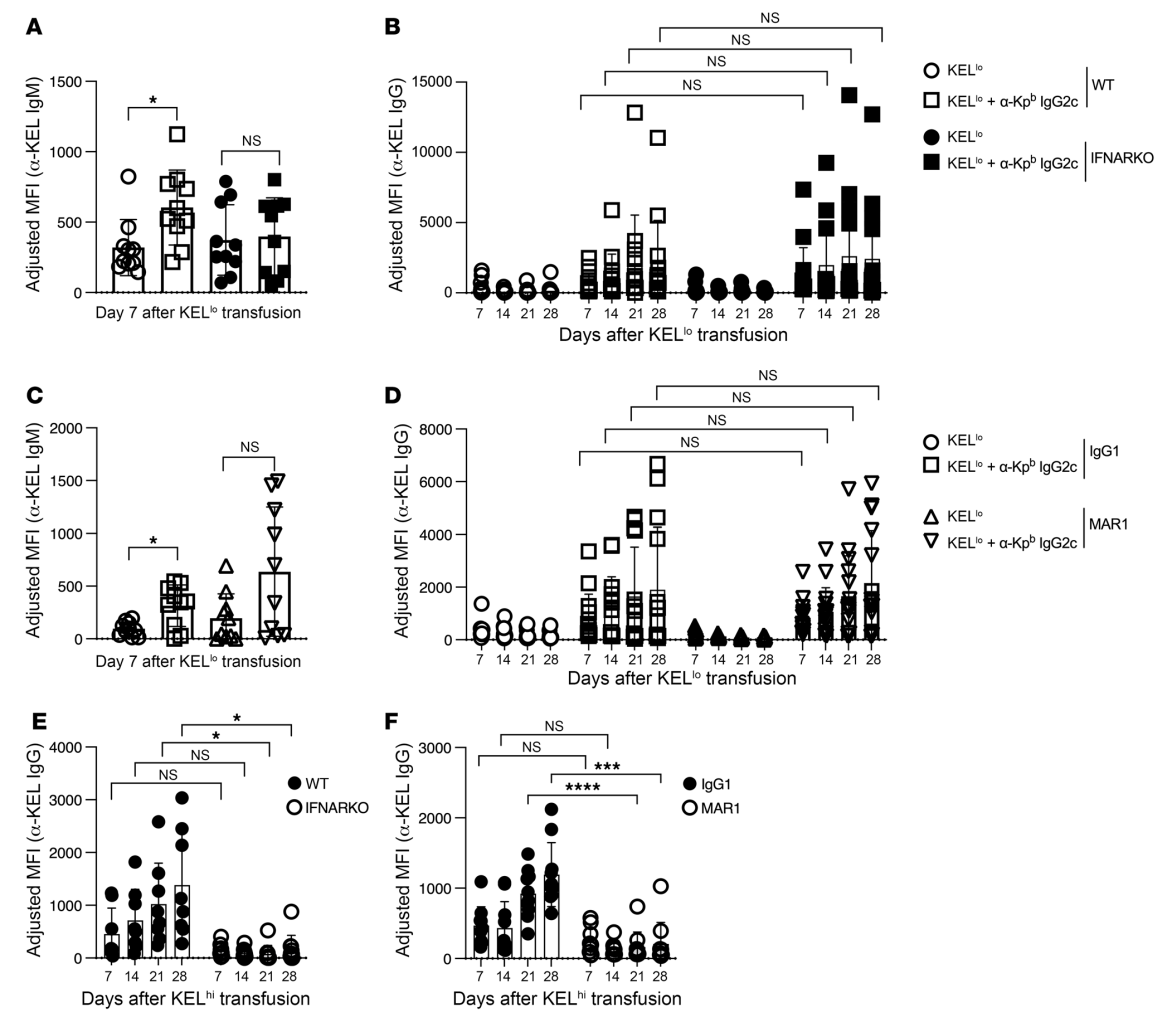
The IFNAR is not required for IgG2c-induced RBC alloimmunization. IgG2c-induced AMIE was measured at the IgM and IgG levels in recipients with a targeted deletion of the IFNAR (**A** and **B**) and in WT recipients treated with an IFNAR-blocking antibody (MAR1) (**C** and **D**). Poly(I:C)–enhanced RBC alloimmunization was decreased in both IFNAR-KO mice (**E**) and WT mice treated with an IFNAR-blocking antibody (**F**), confirming the known effects of disrupting or blocking the IFNAR in other RBC alloimmunization settings. In each case, adjusted MFIs were calculated by subtracting the background antibody signal on antigen-negative B6 RBCs from RBCs expressing KEL, as detailed in Methods. Each experiment was repeated a minimum of 3 times, and representative experiments with 10–15 mice per group are shown. NS (*P* > 0.05), *P < 0.05, ***P < 0.001, and ****P < 0.0001, by multiple Mann-Whitney *U* test (**A** and **C**) and repeated-measures 2-way ANOVA with Šidák’s multiple-comparison test (**B** and **D**–**F**).

To test whether the IFNAR is required for IgG2c-induced AMIE, as it is for enhancement of alloimmunization during viral infection and systemic lupus erythematosus–like (SLE-like) disease (22, 23, 25), we used IFNAR-KO mice and WT controls in the general experimental design (Figure 1). We observed no significant difference in the kinetics or magnitude of IgG2c-induced AMIE in IFNAR-KO mice versus WT mice (Figure 3B). To assess whether a compensatory pathway had been induced in IFNAR-KO mice, as can occur as a result of gene deletion, we performed the same study in WT mice in the absence or presence of an IFNAR-blocking antibody (MAR1) or an isotype-matched IgG1 control. We found that MAR1 treatment had no effect on IgG2c-induced AMIE (Figure 3D). Both IFNAR-KO mice and MAR1-treated WT mice lost polyinosinic:polycytidylic acid–induced [poly(I:C)–induced] alloimmunization against KEL-K2^hi^ RBCs (consistent with previous reports; ref. 25) (Figure 3, E and F), confirming that the intended experimental intervention was achieved. IgM levels did not increase in response to anti-Kp^b^ in IFNAR-KO mice, and an insignificant increase was seen in the presence of MAR1, albeit with higher peak values (Figure 3, A and C). Thus, the effect of the IFNAR on AMIE-induced IgM was unclear. Together, these data demonstrate that IgG2c-induced AMIE worked by a mechanism distinct from enhancement of alloimmunization by viral infection (23), virus-like inflammation with poly(I:C) (25), or SLE-like disease (22), none of which occurs if the IFNAR is disrupted or blocked.

### Antibody-mediated immune enhancement requires FcgRs and involves complement C3.

**Figure 4:**
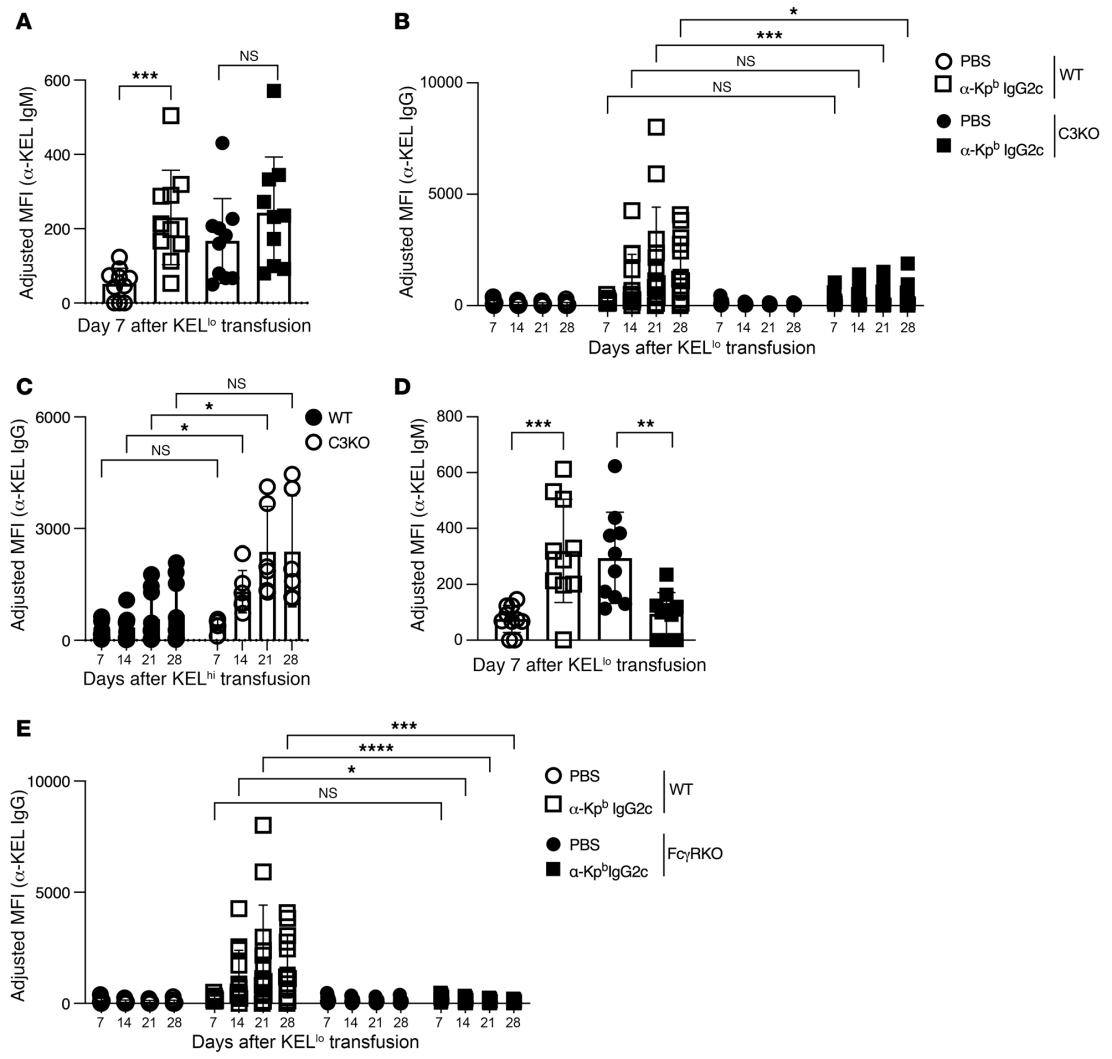
FcγRs are required for and C3 is involved in IgG2c-induced AMIE. (**A** and **B**) Using the experimental design shown in Figure 1A, IgG2c-induced AMIE was decreased at the IgG level in mice with a targeted deletion of the complement C3 gene. (**C**) Consistent with previous reports, the opposite effect of C3 deletion was observed regarding RBC alloimmunization with poly(I:C) and KEL-K2^hi^. (**D** and **E**) IgG2c-induced AMIE was eliminated at the IgM and IgG levels in mice with a deletion of the common γ chain of FcγRs that prevents expression of activating FcγRI, FcγRIII, and FcγRIV, but not inhibitory FcγRII. In all cases, the adjusted MFI was calculated by subtracting the background antibody signal on antigen-negative B6 RBCs from RBCs expressing KEL, as detailed in Methods. Each experiment was repeated a minimum of 3 times with 10–15 mice per group, and representative experiments are shown. *P* > 0.05 (NS), *P < 0.05, **P < 0.01, ***P < 0.001, and ****P < 0.0001, by multiple Mann-Whitney *U* test (**A** and **D**) and repeated-measures, 2-way ANOVA with Šidák’s multiple-comparison test (**B**, **C**, and **E**).

IgG effector function can involve both fixation of complement and ligation of FcgRs. When we applied the experimental design in Figure 1 to mice with a deletion of the complement C3 gene (C3-KO), we detected increased IgM levels in PBS-treated mice (compared with WT mice) but no further enhancement with IgG2c (Figure 4A). In contrast, we observed significantly decreased IgG2c-induced AMIE at the IgG level in C3-KO mice (Figure 4B). Previously, it has been reported that C3-KO mice have an increased alloimmune response to KEL-K2^hi^ (26) [in recipients treated with poly(I:C)] (27, 38), which was confirmed here to validate the experimental system (Figure 4C). Thus, knocking out C3 had the opposite effect on IgG2c-induced AMIE (at the IgG level) when compared with its effect on poly(I:C)-induced alloimmunization. Deletion of the FcgR common γ chain in mice (FcgR-KO mice) resulted in loss of expression of any one of the stimulatory FcgRs (I, III, and IV) but not of the inhibitory FcγII. IgG2c-induced AMIE was eliminated in FcgR-KO mice (Figure 4, D and E). Like in C3-KO mice, we found that baseline IgM was increased in control PBS-treated mice but then decreased in mice treated with anti–Kpb IgG2c (26, 27). The mechanism underlying the baseline changes in IgM in both C3-KO and FcgR-KO is not clear but may represent an important step in the early immune response, including in the absence of AMIE.

### IgG2c induces consumption of KEL-K2^lo^ RBCs by red pulp macrophages.

**Figure 5:**
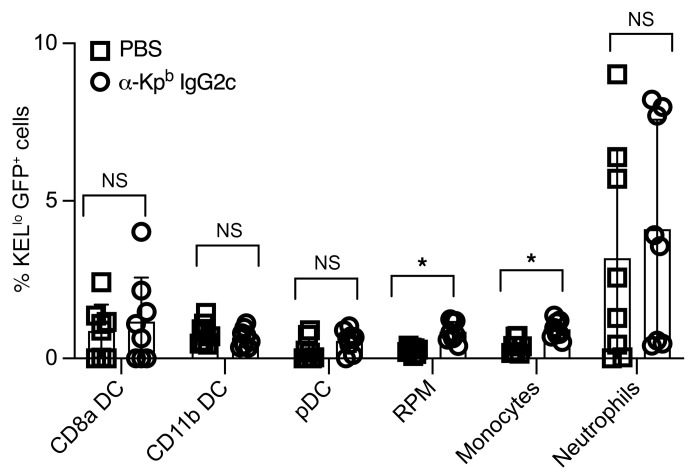
Consumption of KEL-K2^lo^ RBCs by phagocytes during IgG2c-induced AMIE. KEL-K2^lo^ RBCs expressing GFP were used in the experimental design shown in Figure 1. Six hours after transfusion, spleens were harvested, and each of the indicated cell subsets were analyzed for GFP fluorescence (indicating RBC consumption). TER-119^+^ cells were excluded to avoid misinterpreting RBCs stuck to the cell surface as consumption. The only statistically significant increase in consumption was seen in RPMs and monocytes. This experiment was performed over 3 times with 8 mice per group with comparable results. Kinetics were also tested, with no difference in findings (a representative kinetics experiment and representative flow cytometric plots of the consumption by the RPM are shown in Supplemental Figure 2, A and B). *P* > 0.05 (NS) and *P < 0.05, by multiple Mann-Whitney *U* test.

KEL-K2^lo^ mice were crossed with UbiC-GFP mice to generate F1 animals with RBCs expressing both KEL-K2^lo^ and GFP (expression is shown in Supplemental Figure 2A). Consumption of RBCs was assayed by harvesting splenocytes and monitoring the acquisition of GFP fluorescence by different phagocyte populations, using established methods that have been confirmed to identify phagocytosed RBCs (39, 40). We observed a subtle but significant increase in GFP^+^ events in red pulp macrophages (RPMs) (CD11c^–^CD11b^lo^F4/80^+^) (*P* =.001) and also (CD11c^–^CD11b^hi^GR1^+^ monocytes (*P* = 0.002) (Figure 5). We observed no difference in consumption in DC populations (conventional DCs 1 [cDC1], conventional DCs 2 [cDC2], plasmacytoid DCs [pDCs], or neutrophils). The lack of signal in DCs was not due to altered kinetics, as the DCs were analyzed at 15 minutes, 1 hour, 6 hours, and 24 hours after transfusion, with no difference in findings at these time points (data not shown). The IgG2c-mediated increase in RPM consumption peaked by 6 hours and persisted until at least 24 hours after transfusion (Supplemental Figure 2, B and C). The increased GFP signal was not the results of RBCs sticking to the phagocyte surface, as all events were TER119^–^, consistent with the assay previously validated by microscopy (40). Serum obtained from additional mice from the same experiment 21 days after treatment confirmed that AMIE was unaltered by the presence of GFP in the RBCs (Supplemental Figure 2D).

Frozen sections of spleens harvested at the 1-hour time point were examined by fluorescent microscopy to assess RBC localization. We detected no difference in GFP signal between mice that received IgG2c versus those that received PBS (data not shown). This was not due to a failure of the assay, as localization in the MZ was observed when using RBCs that had robust clearance (KEL-K2^hi^ GFP RBCs, data not shown). These findings were consistent with the low levels of RBC clearance of KEL-K2^lo^ RBCs (see Figure 2C).

### Antibody-mediated immune enhancement is a CD4^+^ T cell–dependent process.

**Figure 6:**
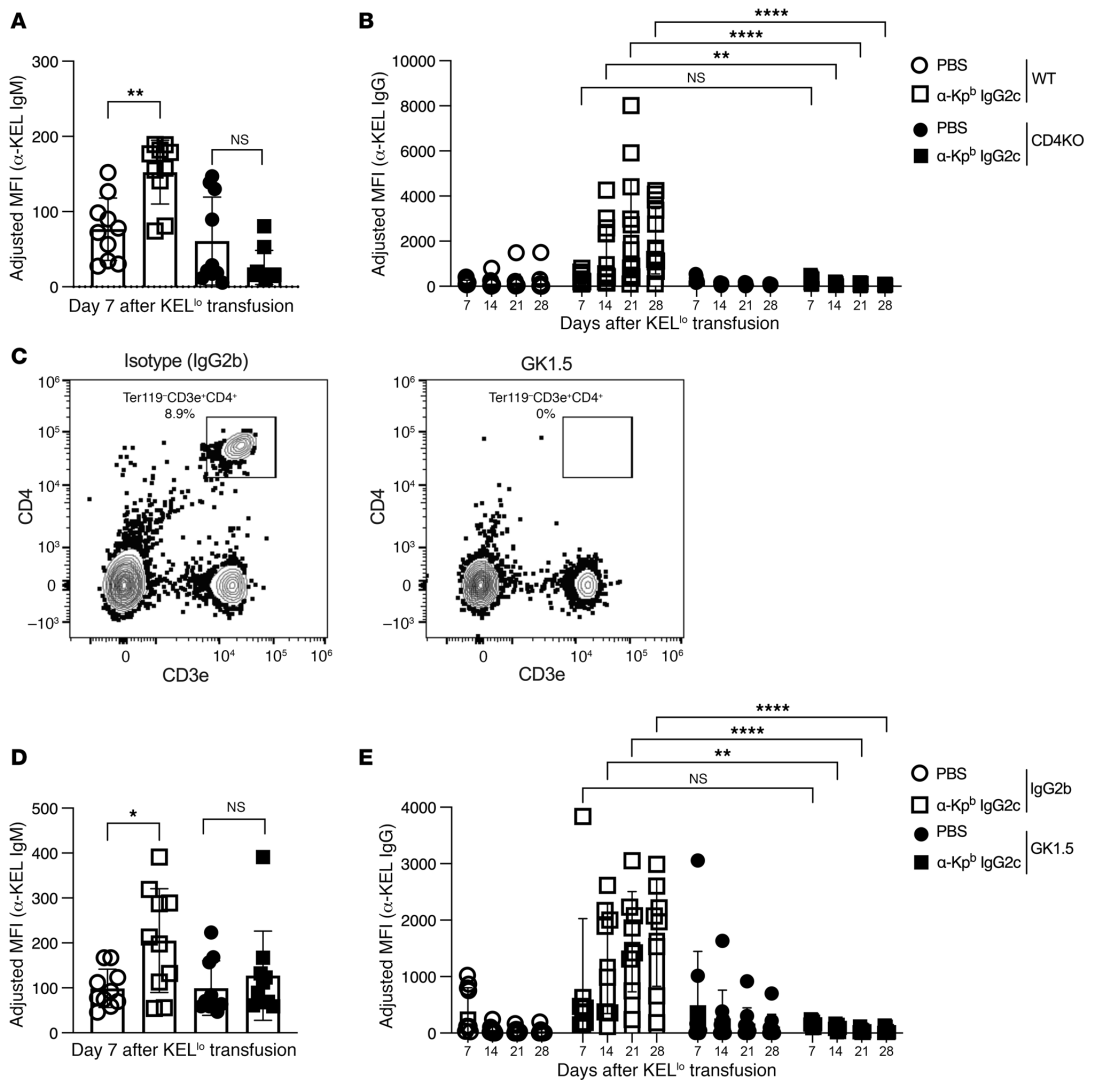
CD4^+^ T cells are required for IgG2c-induced RBC alloimmunization. (**A** and **B**) Using the experimental design shown in Figure 1A, IgG2c-induced RBC alloimmunization was prevented at both the IgM and IgG levels in mice with a targeted deletion of CD4. (**C**) Similarly, treatment of mice with a CD4^+^ T cell–depleting antibody (GK1.5), which was confirmed by flow cytometry (**D** and **E**) prevented IgG2c-induced AMIE at the IgM and IgG levels. Robust IgG2c-induced AMIE was still observed in mice treated with an isotype matched control for GK1.5, ruling out nonspecific effects of IgG2b injection. The adjusted MFI was calculated by subtracting background antibody signal on antigen-negative B6 RBCs from RBCs expressing KEL, as detailed in Methods. Each experiment was repeated a minimum of 3 times with 10–15 mice per group and representative experiments are shown. *P* > 0.05 (NS), *P < 0.05, **P < 0.01, and ****P < 0.0001, by multiple Mann-Whitney *U* test (**A** and **E**) and repeated-measures, 2-way ANOVA with Šidák’s multiple-comparisons test (**B** and **E**).

Alloimmunization against transfused RBCs expressing KEL-K2 can occur by either CD4^+^ T cell–dependent or –independent pathways, depending on the KEL-K2 copy number (26, 36, 41, 42). Because KEL-K2^lo^ does not induce significant anti–KEL IgG in other settings (37), the CD4^+^ T cell dependence has not been evaluated. We observed no IgG2c-induced AMIE at the IgM or IgG levels in CD4-KO mice compared with WT controls (Figure 6, A and B). As an alternative approach, CD4^+^ T cells were depleted from WT mice using a well-known monoclonal depleting antibody (clone GK1.5), and depletion of CD4^+^ T cells after treatment was confirmed by flow cytometry using a fluorescent anti-CD4 antibody (clone RM4) that was not blocked by GK1.5 (Figure 6C). We observed no IgG2c-induced AMIE in CD4^+^ T cell–depleted mice at the IgM or IgG levels (Figure 6, D and E). The effect of GK1.5 was specific, as enhancement still occurred in mice injected with an isotype matched control for the CD4^+^ T cell–depleting antibody (IgG2b). Together, these data indicate that CD4^+^ T cells were required for IgG2c-induced AMIE.

### MZ B cells play a role in IgG2c-mediated enhancement.

**Figure 7:**
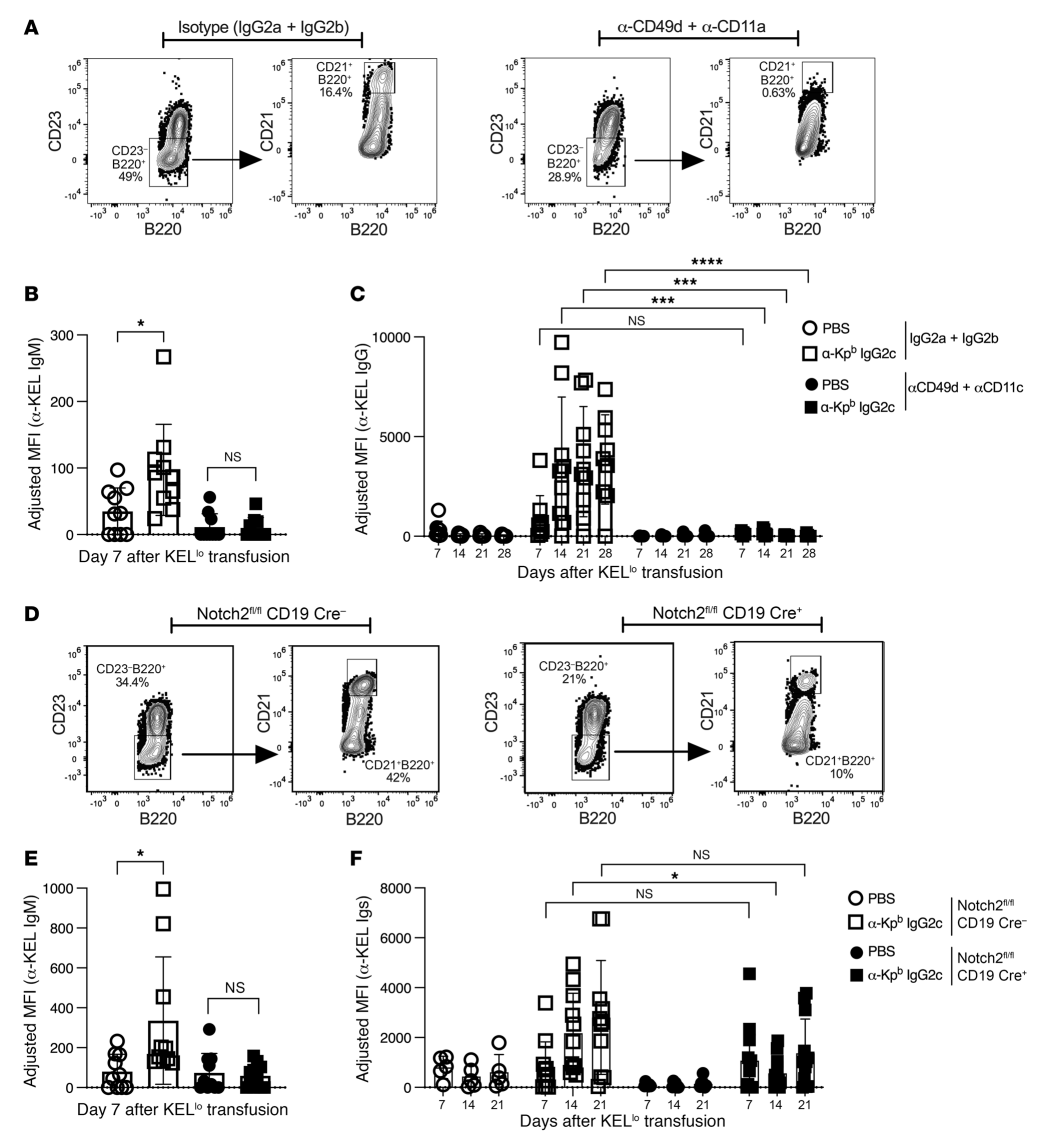
MZ B cells are required for IgG2c-induced RBC alloimmunization. Using the experimental design shown in Figure 1A, WT mice were treated with an antibody cocktail known to deplete MZ B cells (anti-CD49b plus anti-CD11c). (**A**) Depletion was confirmed by flow cytometry, and (**B** and **C**) treatment prevented IgG2c-induced AMIE at both the IgM and IgG levels. Robust IgG2c-induced AMIE still occurred with injection of isotype-matched controls, ruling out nonspecific effects of injection of IgG2a and IgG2b (see Supplemental Figure 3A). (**D**) As reported, mice with a B cell conditional deletion of Notch2 had decreased numbers of MZ B cells and (**E** and **F**) had significantly decreased IgG2c-induced AMIE at the IgM and IgG levels**.** The adjusted MFI was calculated by subtracting the background antibody signal on antigen-negative B6 RBCs from RBCs expressing KEL, as detailed in Methods. Each experiment was repeated a minimum of 3 times with 5–12 mice per group, and representative experiments are shown. *P* > 0.05 (NS), *P < 0.05, ***P < 0.001, and ****P < 0.0001, by multiple Mann-Whitney *U* test (**B** and **E**) and repeated-measures and 2-way ANOVA with Šidák’s multiple-comparison test (**C** and **F**).

It has previously been reported that MZ B cells are required for both CD4^+^ T cell–dependent and –independent RBC alloimmunization (20, 21). To test whether MZ B cells are required for IgG2c-induced AMIE, MZ B cells were depleted by standard methods using a mixture of anti-CD49 and anti-CD11c antibodies (21). Depletion of MZ B cells was confirmed by flow cytometry (Figure 7A), and IgG2c-induced AMIE was found to be eliminated at both the IgM and IgG levels (Figure 7, B and C). An isotype-matched control for the MZ-depleting cocktail (IgG2a and IgG2b) had no effect (Figure 7C). There was also no difference between mice receiving isotype-matched control and those that did not receive an antibody injection (Supplemental Figure 3A).

Although anti-CD49 and anti-CD11c antibodies depleted MZ B cells, the depletion was not entirely MZ B cell specific, and other cell populations were affected. To elicit a more MZ B cell–specific decrease, we used *Cd19-CrexNotch2^fl/fl^* mice, which are known to have a developmental defect in MZ B cell generation. Consistent with previous reports, MZ B cells were decreased but not eliminated (Figure 7D). *Cd19-CrexNotch2^fl/fl^* mice had decreased IgG2c-induced AMIE at both the IgM and IgG levels, the latter being significant at day 14 (*P* = 0.04) (Figure 7, E and F). To confirm the phenotype, *Cd19-CrexNotch2^fl/fl^* mice were transfused with KEL-K2^med^ RBCs and, as previously reported (27), had decreased alloimmunization (Supplemental Figure 3B). The decrease (but not elimination) of AMIE in *Cd19-CrexNotch2^fl/fl^* mice was consistent with the decrease (but not elimination) of MZ B cells. Together, these data indicate that MZ B cells played a role in IgG2c-enhanced RBC alloimmunization.

### IgG2c-induced AMIE results in no significant increase in GC B cells.

**Figure 8:**
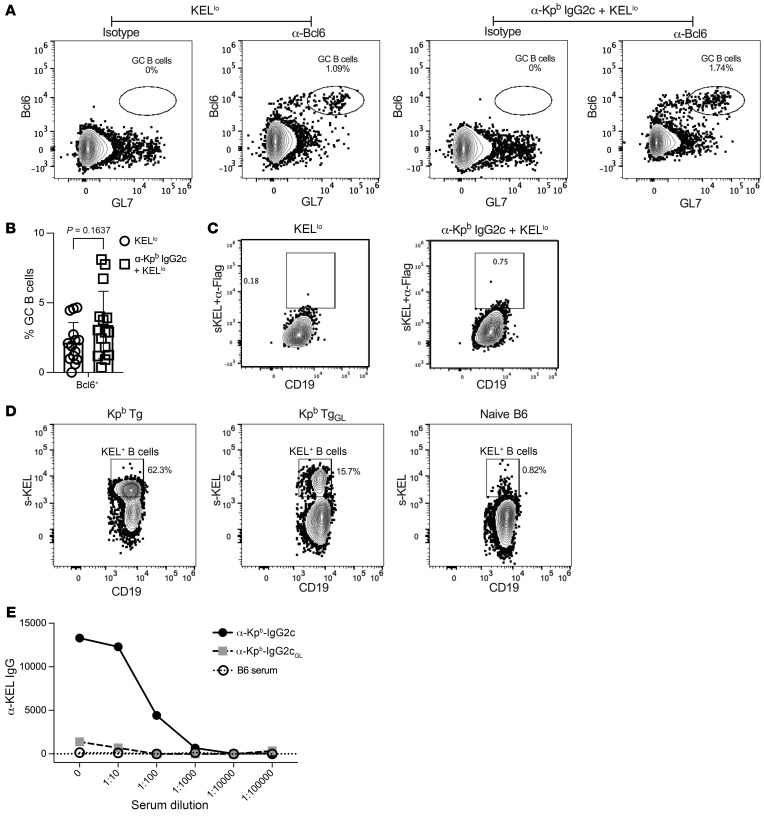
IgG2c-induced AMIE does not significantly increase germinal center B cells. Using the experimental design shown in Figure 1A, a small increase in germinal center B cells (BCL6^+^, GL7^+^) was observed in IgG2c-induced AMIE, but it did not achieve statistical significance (*P* = 0.16), thus it was concluded that there was no difference. (**A**) Representative flow plots are shown, and (**B**) the combined results from 3 experiments with 3–5 mice per group are shown. sKEL antigen was used as a stain for antigen-specific GC B cells. (**C**)Although a subtle increase in staining was observed, it was deemed insignificant compared with positive control staining in cells from BCR B cell–transgenic mice expressing either a high-affinity (**D**, middle panel) or low-affinity (**D**, right panel) BCR for sKEL. (**E**) Decreased affinity of lower-affinity anti-Kp^b^ IgG (IgG2cGL) was confirmed by titrating purified recombinant antibody with KEL-K2 RBCs by flow cytometry. This experiment was repeated 3 times with similar results, and representative flow plots are shown. *P* values were calculated using a multiple Mann-Whitney *U* test.

No appreciable expansion of antigen-specific GC B cells has been detected during primary alloimmunization against either of 2 different RBC alloantigens in mice (20, 24, 31, 32). To test whether IgG2c-induced AMIE involved GC expansion, we stained splenocytes with anti-BCL6 and anti-GL7 antibodies 14 days after transfusion when IgG alloantibodies were high in serum. A clear population of GC B cells (IgD^–^BCL6^+^GL7^+^) was detected (Figure 8A); anti-BCL6 staining was specific, as no staining was seen with an IgG1 isotype–matched control. Although there was a trend toward an increase with IgG2c treatment, with a wide distribution among mice (Figure 8B), no statistically significant difference in total GC B cells was seen in mice treated with IgG2c anti-Kp^b^ prior to transfusion (Figure 8B, *P* = 0.16).

In order to test whether there was a change in KEL-reactive GC B cells, we developed an antigen-specific stain by inducing expression of the extracellular domain of human KEL as a recombinant soluble protein (sKEL) (43). The sKEL maintains native epitopes, as previously reported (43), and is recognized both by monoclonal anti-Kp^b^ antibody and also polyclonal anti–KEL IgG in the serum of immunized mice (as confirmed by ELISA; data not shown). This allowed staining of KEL-specific B cells with sKEL followed by staining with fluorescently labeled anti-KEL. We detected a small population of weakly sKEL-reactive B cells after IgG2c treatment (Figure 8C, right panel). However, to interpret these data, it is necessary to carefully characterize the sensitivity of the sKEL staining reagent.

As part of the ongoing refinement of immunological tools for the study of RBC alloimmunization, we generated a B cell receptor–transgenic (BCR-transgenic) mouse by knocking the heavy and light chain variable domains of anti-Kp^b^ (clone PUMA4) into the V_H_ and L_H_ loci, respectively (Kp^b^Tg). sKEL reacted with approximately 62% of B cells from the Kp^b^Tg mice with trace background staining of WT B6 B cells (Figure 8D). The 38% non-sKEL-reactive B cells were due to background recombination of the WT Ig locus, as these mice were not on a RAG-KO background. These data demonstrate the capacity to detect KEL-specific B cells with the sKEL stain. Furthermore, these data suggest that the weak shift seen in Figure 8C was not significant. However, one caveat to this interpretation is that the Kp^b^-transgenic mouse was generated with a sequence from an affinity-matured antibody, which is expected to have a far higher affinity than do antibodies at the beginning of a primary humoral response.

To address the above caveat, we generated a second mouse in which the V_H_ and L_H_ domains were reverted to germline V and D sequences, to acquire a low-affinity BCR-transgenic mouse (Kp^b^Tg_GL_). A recombinant antibody with the germline sequence (anti-Kpb IgG2c_GL_) was expressed and compared with the original antibody by titrating with KEL-K2 RBCs and monitoring binding by flow cytometry (Figure 8E). As predicted, anti-Kpb IgG2c_GL_ bound to KEL-K2 RBCs but with a greatly diminished capacity compared with the original antibody, consistent with a substantially lower affinity. sKEL also stained B cells from the Kp^b^Tg_GL_ mouse, and although the percentage of stained cells decreased, the intensity of staining was similar. The decreased percentage was likely due to the ability of sKEL to bind to B cells expressing only the transgenic heavy chain, but with a WT light chain from Kp^b^Tg mice that required both transgenic chains from the Kp^b^Tg_GL_ mice. Together, these data demonstrate that the sKEL staining could detect even low-affinity B cells and that the subtle staining of GC B cells in mice undergoing IgG2c-induced AMIE was not consistent with BCR binding of even low-affinity BCRs.

## Discussion

The current report elucidates mechanistic properties of IgG2c-induced AMIE that are distinct from RBC alloimmunization in other settings. The IFNAR has emerged as an essential receptor for RBC alloimmunization (both CD4^+^ T cell dependent and independent), both at baseline and when enhanced by inflammation (viral infection or lupus-like pathology) (22–25). Although characterized mostly in murine systems, viral infection has translated as an independent risk factor for alloimmunization in humans (44) and an “interferon signature” is associated with human pathologies in which alloimmunization is increased (45–48). In addition, complement C3 has emerged as an inhibitor of RBC alloimmunization (26, 27). In stark contrast to the above mechanisms of RBC alloimmunization, the IFNAR is not required for IgG2c-induced AMIE, and C3 contributes to (rather than inhibits) alloantibody formation. Also, unlike other forms of RBC alloimmunization, FcgRs are required in IgG2c-induced AMIE (31, 49). However, mechanisms of AMIE share a general requirement for MZ B cells with other pathways of RBC alloimmunization (20, 21). Also, like other mechanisms of RBC alloimmunization, we observed no significant increase in antigen-specific GC B cells, although a small population of sKEL weakly–reactive GC B cells.

A general model of IgG2c-induced AMIE emerges by combining the current findings with published observations (31, 37, 49). It has been shown that IgG2c-induced AMIE is eliminated with either conditional deletion of FcgRs on CD11c^+^ cells (31) or disruption of FcgRIV (49), suggesting that AMIE works through FcgRIV on DCs. In a separate antigen system, IgG2c-induced AMIE has been shown to cause increased CD4^+^ T cell activation and expansion (31), consistent with the requirement for CD4^+^ T cells in the current report. In the current study, we observed no increased consumption of RBCs by DCs, only increases in RPMs and monocytes. RPMs or monocytes could, in theory, serve as primary antigen-presenting cells (APCs). However, in other RBC alloimmunization systems, it has been shown that DCs, but not RPMs, are capable of primary activation of CD4^+^ T cells and that a small population of DCs (bridging channel DCs) are required (50). Given that the current study evaluated RBCs with very low levels of antigen and of clearance, we could not assess the sensitivity of the assay to visualize consumption by DCs. As such, our working model encompasses the observations of DCs from other related AMIE systems (31, 49). However, any APC subset could function equally well in the proposed model. There are several possibilities for how the patterns observed with IgM fit into a working mechanism, such as alterations in class switching. Although the current studies do not elucidate the direct role of IgM, the potential importance of IgM effects should not be discounted. Future studies will be required to investigate the role of IgM.

The overall model is that RBC-bound IgG2c promotes DC consumption through ligation of FcgRIV. In both humans and mice, ligation of FcgRs on DCs is known to (a) increase antigen uptake and presentation, (b) increase costimulatory molecule expression on the DC (signal 2), and (c) increase secretion of cytokines that promote CD4^+^ T cell activation and differentiation (signal 3) (51, 52). Indeed, antigen-antibody complexes result in both greatly increased antigen presentation and also APC maturation compared with soluble antigen (53, 54). IgG2c also activates the complement cascade, generating C5a and also fixing iC3b on the RBC surface, both of which are known to enhance DC function (55, 56). Expanded CD4^+^ T cells then go on to help B cells differentiate into plasmablasts. It is unclear if the plasmablasts are the source of plasma cells (as is seen for predominantly extra follicular (EF) antigens) (57) or if GCs are involved; however, if GCs are involved they are more subtle than what is typically observed for more robust antigens (57).

With regard to the role of antigen modulation in immune regulation by anti-RBC alloantibodies, it has repeatedly been speculated, or stated as a fact, that AMIS occurs because antigen modulation redirects antigens away from immunogenic processing and presentation (27, 58–62). In each of these studies, the actual observation was that antigen modulation preceded AMIS, but the causal role of antigen modulation was never tested. The findings in the current study (combined with previous publications) show that antigen modulation also proceeded AMIE in 5 different alloantigen systems (HOD, KEL-K2^hi^, KEL-K2^med^, KEL-K2^lo^, and human glycophorin A [hGPA]) (31, 37, 58). Thus, there is just as much evidence of antigen modulation playing a central role in AMIE as in AMIS. This has led to the speculation that antigen modulation causes both AMIE and AMIS but that the antigen is directed to immunogenic or nonimmunogenic pathways depending on various factors, such as the ratio of RBCs to antibodies (58). This is, of course, a plausible explanation for the seemingly contradictory findings. However, the findings can also be explained if antigen modulation is just a general property of antibody-binding RBCs that causes neither AMIE nor AMIS.

Indeed, Xu and Heyman correctly pointed out additional observations that are incompatible with a causal role of antigen modulation in either AMIS or AMIE (63). First, AMIE occurs with regard to a third-party antigen expressed on the same RBCs but not recognized by the injected antibody (31, 63). This observation is incompatible with antigen modulation, unless the third-party antigen is also modulated, but this was shown not be the case (31, 63). AMIS has been shown to not extend to different antigens on the same RBCs in mice (59), however, the opposite has been observed in a human trial, in which an injection of plasma containing anti-KEL1 suppressed responses to RhD when volunteers were transfused with RBCs expressing both KEL1 and RhD alloantigens (64). Moreover, it has been shown that antibody responses to the specific epitope recognized by IgG can be suppressed, while enhancing immunization against another epitope on the same protein or protein-antibody complex (65–67). Each of these observations is incompatible with antigen modulation causing AMIS or AMIE.

To the best of our knowledge, no direct experimentations have been able to formally test the causal role of antigen modulation in either AMIS or AMIE. This is because there is no known method to selectively remove antigen modulation but leave all other processes intact. Antigen modulation seems to be a general property of antibodies against RBCs and has been observed in at different least 9 different blood group systems in humans in terms of alloantibodies, autoantibodies, and therapeutic antibodies (68, 69). In light of all of the above evidence, we agree with Xu and Heyman (63) and offer the interpretation that antigen modulation is a general property of anti-RBC alloantibodies that is coincidental to immunomodulation and does not play a causal role in either AMIE or AMIS. Resolution of this question will require a method to isolate antigen modulation as an independent variable.

Why MZ B cells are required for IgG2c-mediated AMIE is unclear. To the best of our knowledge, there have been no findings of RBC alloimmunization (by any pathway) in which MZ B cells have been shown not to be required — suggesting that MZ B cells may be essential to RBC alloimmunization (in general) and therefore an attractive therapeutic target. The requirement for MZ B cells is likely due to one of their specialized functions of (a) trafficking antigen into follicles, (b) serving as a primary APC, or (c) giving rise to antibody-secreting B cells (70). RBC alloimmunization against transfused murine RBCs (HOD system, in which transgenic mouse RBCs express a fusion protein made of HEL, OVA, and human Duffy blood group antigen) is not decreased in mice with a targeted deletion of sphingosine 1 kinase, which have a defect in MZ B cell trafficking, suggesting that antigen trafficking is not required (20).

There are several practical ramifications of IgG-mediated AMIE that may explain its evolution. AMIE entails existing antibodies against antigens for which a CD4^+^ T cell response has not yet occurred. Such a pathway could offer an advantage by augmenting primary immune responses to pathogens related to previously encountered microbes that cross-react with previously formed antibodies. Alternatively, early IgG coming from CD4^+^ T cell–independent activation of plasmablasts could augment subsequent CD4^+^ T cell help as part of a primary immune response. Finally, passive immunization of newborns by maternal antibodies could provide “virtual CD4^+^ T cell memory,” if maternal antibodies promote CD4^+^ T cell–based immunity when a newborn is infected with a microbe that maternal antibodies recognize. These possibilities are not mutually exclusive, and ongoing work should assess these scenarios.

It has been demonstrated that transfusion-induced IgG can exist at levels that cause clearance of transfused RBCs but are not detectable by serological assays (71). Accordingly, transfusions may be inadvertently given to patients who have subclinical IgG that is reactive against antigens on the transfused RBCs, which could cause robust humoral alloimmunization. As such, subclinical anti–RBC IgG not detected during patient screening may cause AMIE to another antigen expressed by transfused RBCs. Thus, AMIE may contribute to the responder/nonresponder divide (72). However, we are unaware of this question being tested.

As with all model systems, there are limitations with the current studies, and there is always the risk that the mechanisms elucidated here may not translate between species. However, the phenomenon of AMIE by IgG against RBCs has been observed in both mice and humans. Murine IgG2c is a variant of the mouse *IgG2a* gene (73), which most closely lines up with human *IgG1* with regard to FcgR affinity and complement fixation (74). The current studies with our system advance our understanding of the mechanisms of AMIE induced by IgG2c (in particular) and our overall understanding of immune regulation by anti-RBC antibodies (in general) and, to our knowledge, define the first pathway of enhancing RBC alloimmunization that does not require the IFNAR and to which C3 contributes.

## Methods

### Sex as a biological variable.

Only female animals were used for these studies, with the rationale that immunomodulatory therapy with antibodies against blood group antigens (i.e., anti-RhD) is currently only used in female patients, as its effects only manifest in pregnancy. However, the current studies also contain general basic mechanistic elucidations that may be relevant to immunobiology in males. Thus, although the findings are most therapeutically relevant to females, the biological relevance may apply to males as well.

### Mice.

C57BL/6J, Fcer1g-KO (FcgR), CD4-KO (B6.129S2-*Cd4^tm1Mak^*/J), IFNAR1-KO (B6.129S2-Ifnar1tm1Agt/Mmjax), complement C3–KO (B6.129S4-*C3^tm1Crr^*/J), UBI-GFP [C57BL/6-Tg(UBC-GFP)30Scha/J], CD19Cre [B6.129P2(C)-Cd19tm1(Cre)Cgn/J; H-2b], and Notch2flx (B6.129S-Notch2tm3Grid/J; H-2b) mice were purchased from The Jackson Laboratory. KEL-K2^hi^, KEL-K2^med^, KEL-K2^lo^, and mCherry transgenic mice were generated on a B6 background as previously described (36, 75, 76). Each KEL-K2 mouse expressed the same variant of the human KEL glycoprotein (K2^+^, Kp^b+^, Js^b+^) but with a different RBC copy number and different integration sites (41). In all cases, KEL-K2^lo^ was the RBC type being studied with regard to the mechanism of the phenomenon under investigation (i.e., enhancement by IgG2c). KEL-K2^med^ and KEL-K2^hi^ were only used as controls to confirm that the experimental interventions were working in a manner consistent with previous reports. KEL-K2^hi^ was used as a target for measuring alloantibodies, as it has the highest level of K2 expression and is thus the most sensitive target. An initial description of 2 BCR-transgenic mice expressing high- or low-affinity anti-Kp^b^ is included in this study, and these mice were used as controls for staining of KEL-specific B cells during primary alloimmunization. The use of these animals to study B cell responses to RBC alloantigens is outside the scope of the current work; however, additional details on the generation of these animals are included in Supplemental Methods. All mice were used at 8–12 weeks of age. Antibody and RBC infusions were given intraperitoneally and/or intravenously, respectively.

### Generation, expression, and purification of monoclonal antibody switch variants.

Expression of PUMA3 (anti-Js^b^) IgG2b and PUMA4 (anti-Kp^b^) IgG2c was induced as previously described by cotransfecting expression vectors for heavy and light chains into either CHO cells or HEK cells and purifying them to homogeneity using protein A/G sepharose (37). Low-affinity PUMA4 was generated using site-directed mutagenesis to revert the V and D domains to a germline sequence (i.e., anti-Kp^b^_GL_) and expressed in the same manner. Details on anti-Kp^b^_GL_, as well as on the entire DNA/RNA sequences for the heavy and light chains of PUMA4, are provided in the Supplemental Methods.

### Cellular depletion, receptor blockade, and RBC transfusion.

CD4^+^ T cells were depleted by 2 intraperitoneal injections of 250 μg monoclonal anti–mouse CD4 (clone GK1.5, Bio X Cell) 4 and 2 days prior to transfusion, and depletion was confirmed by staining peripheral blood with anti–mouse CD4 antibody (clone RM4, BioLegend). Control mice received the same dose and injection schedule of an isotype-matched control IgG2b (clone LTF2, Bio X Cell). MZ B cells were depleted by treating B6 recipients 4 and 2 days prior to transfusion with intraperitoneal injections of 100 μg monoclonal anti–mouse CD11a antibody (clone M17/4, Bio X Cell) and 100 μg monoclonal anti–mouse CD49d antibody (clone PS/2, Bio X Cell); control mice received a combination of isotype-matched IgG2a and IgG2b control antibodies (clones LTF2 and 2A3, Bio X Cell). Splenocytes were stained with antibodies against CD45R, CD23, and CD21 to assess and confirm MZ B cell (B220^+^CD23^−^CD21^hi^) depletion. For IFNAR blocking, mice were treated with 600 μg MAR1-5A3 (BE0241) or IgG1 (BE0083) isotype control antibody on days 4 and 2 prior to transfusion. Mice were infused (as indicated in the figures) with 1 μg anti-KEL IgG2c (Puma4, WT or mutant) 2 hours prior to KEL-K2^lo^ RBC transfusion. In some cases, as controls for known biology, poly(I:C) was given 2 hours prior to transfusion of KEL-K2^hi^ or KEL-K2^med^ was given alone. RBC donor whole blood was collected in citrate phosphate dextrose adenine 1 (CPDA-1) and washed with PBS. Recipients were then transfused intravenously by lateral tail vein injection with 50 μL packed RBCs diluted in PBS to a total volume of 250 μL.

### Determination of post-transfusion circulation, antigen expression, C3 deposition, and antibody binding to RBCs and measurement of humoral immune responses to RBC alloantigens.

Mice were transfused with a 1:1 mixture of DiO-labeled KEL-K2^lo^ and mCherry RBCs intravenously via lateral tail vein injections and were bled at the indicated time points by retro-orbital bleeding and/or tail bleeding. RBC survival was measured by the ratio of DiO-KEL-K2^lo^ RBCs to mCherry control RBCs, which was normalized to the same ratio of the pretransfusion mix (input). The amount of KEL-K2^lo^ antigen on transfused RBCs (e.g., measurement of antigen modulation) was measured by staining with anti-KEL IgG2b (clone Puma3-IgG2b) followed by goat anti–mouse IgG2b APC-Cy7. To determine the amount of antibody bound to RBCs in vivo, RBCs were stained with goat anti–mouse Ig (IgG2c) APCs (equivalent of a DAT). Complement deposition on RBCs was measured by an anti-C3 antibody that recognizes all C3 forms (Cedarlane Labs, biotinylated clone RmC11H9). For each of the above (PUMA3, anti-IgG2c, and anti-C3), staining was assessed after gating on DiO^+^ RBCs to isolate the infused KEL-K2^lo^ cell population.

Alloantibody responses in transfusion recipients were measured by a flow cytometric crossmatch assay, as described above, using fluorescently labeled secondary antibodies specific for either IgM or total IgGs (Southern Biotech). In brief, sera from transfused mice were collected at the indicated time points and incubated with target RBCs expressing either KEL-K2^hi^ (antigen^+^ RBCs) or B6 (antigen^–^ RBCs) followed by the fluorescently labeled secondary antibody. The antigen-specific response (i.e., adjusted MFI) was determined by subtracting the MFI of B6 RBCs (background) from the MFI of KEL-K2^hi^ RBC targets. All flow cytometry was performed on an Attune NxT Cytometer (Thermo Fisher Scientific) and analyzed using FlowJo software.

### Analysis of mouse splenic APC subsets by flow cytometry.

Mice were transfused with KEL-K2^lo^.GFP RBCs by lateral tail vein injection, spleens were harvested at the indicated time points, and single-cell suspensions of splenocytes were prepared. Following RBC lysis, spleens were treated with Fc block (anti–mouse CD16/CD32, clone 2.4G2) and surface stained with antibodies against the different splenic immune cell populations. CD3e (clone 17A2), CD19 (clone ID3), NK1.1(clone PK136), TER119 (clone TER 119), CD11b (clone M1/70), CD11c (clone N418), CD8a (clone 53-6.7), F4/80 (clone BM8), CD115 (CSF-1R, clone AFS98), Ly-6G/Ly-6C (GR-1, clone RB6-8C5), and PDCA-1 (CD137, BST2, clone 927) were purchased from BioLegend, and anti–mouse CD16/CD32 was purchased from BD Biosciences. Flow cytometric data were acquired on an Attune NxT Cytometer (Thermo Fisher Scientific) and analyzed with FlowJo. RBC consumption was determined by GFP expression in each subset, and signal from RBCs stuck to the cell surface was ruled out by gating on only TER-119 negative events as per established and validated protocols (40). The gating strategy for each cellular subset is shown in Supplemental Figure 4.

### Statistics.

Statistical analysis was performed using an unpaired Mann Whitney *U* test and a repeated measures, 2-way ANOVA with Šidák’s multiple-comparison test. *P* values of less than 0.05 were considered statistically significant. All data are presented as the mean ± SEM.

### Study approval.

All animal studies were carried out according to approved IACUC protocols of the University of Virginia.

### Data availability.

The corresponding author will provide all requested materials, data sets, and protocols, without restriction, upon request. See the Supporting Data Values file for all data shown.

## Author contributions

AJ and JCZ designed the studies. AJ, JBC, AMH, and TP carried out the experiments. AJ, JCZ, KEH, and CJL interpreted the data. AJ and JCZ drafted the manuscript, and all authors participated in its writing and editing.

## Supplementary Material

Supplemental data

Supporting data values

## Version 1 04/15/2024

Electronic publication
