# Supplementary material for: Complement C3 and marginal zone B cells promote IgG-mediated enhancement of RBC alloimmunization in mice
Source: J Clin Invest. 2024 Apr 15;134(8):e167665. doi: 10.1172/JCI167665 (PMC11014669; doi:10.1172/JCI167665)
Supplement: Supplemental data [file jci-134-167665-s221.pdf]

Supple Figure 1.

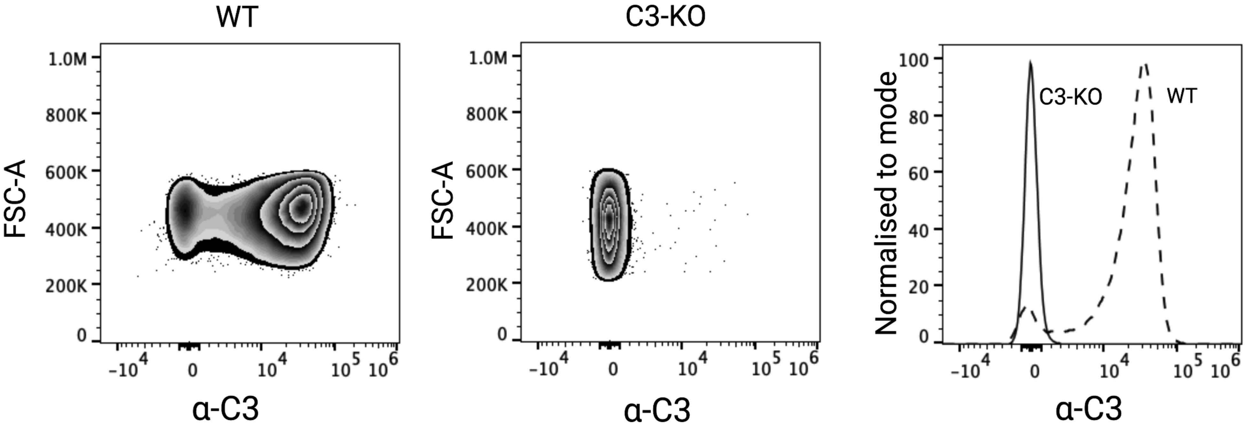

Supple Figure 2.

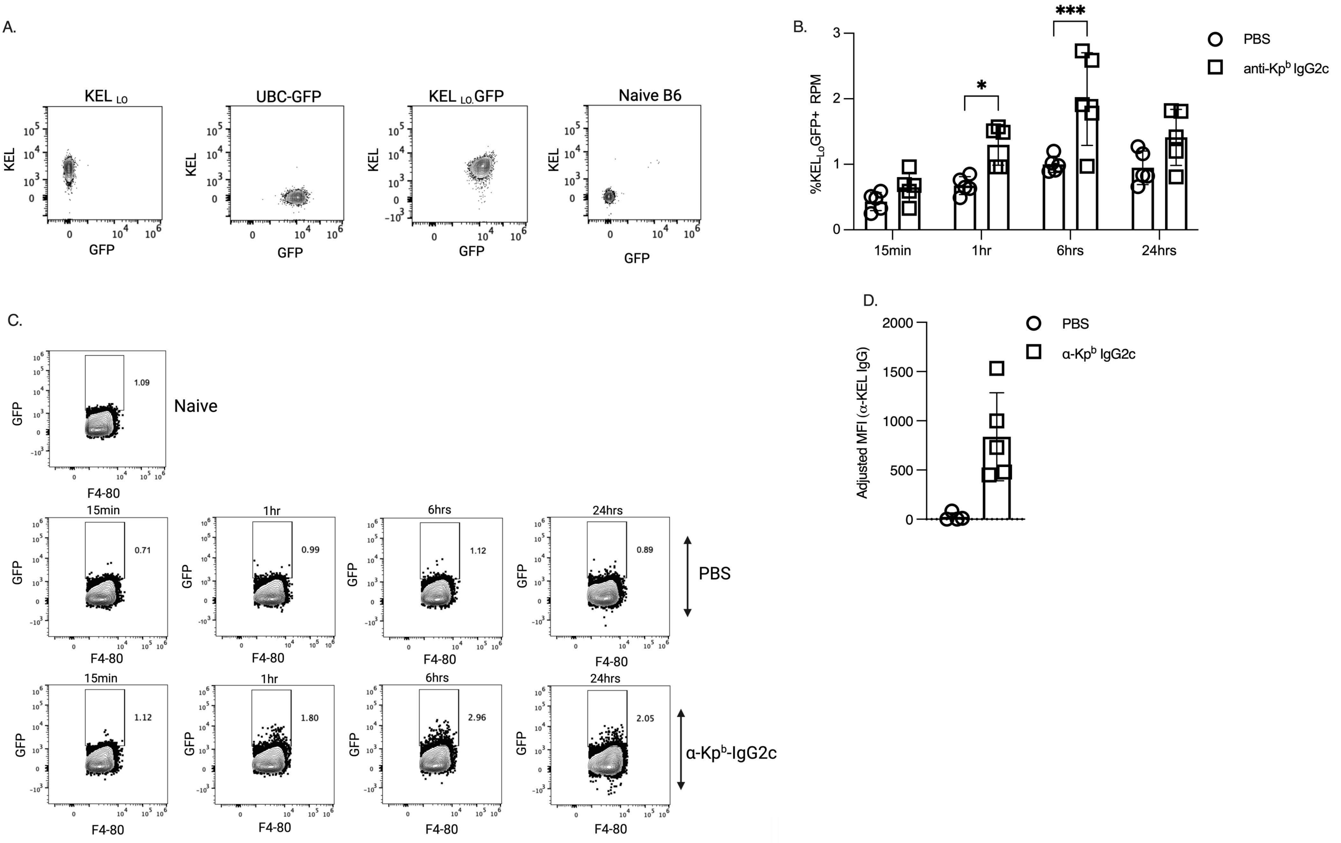

Supple Figure 3.

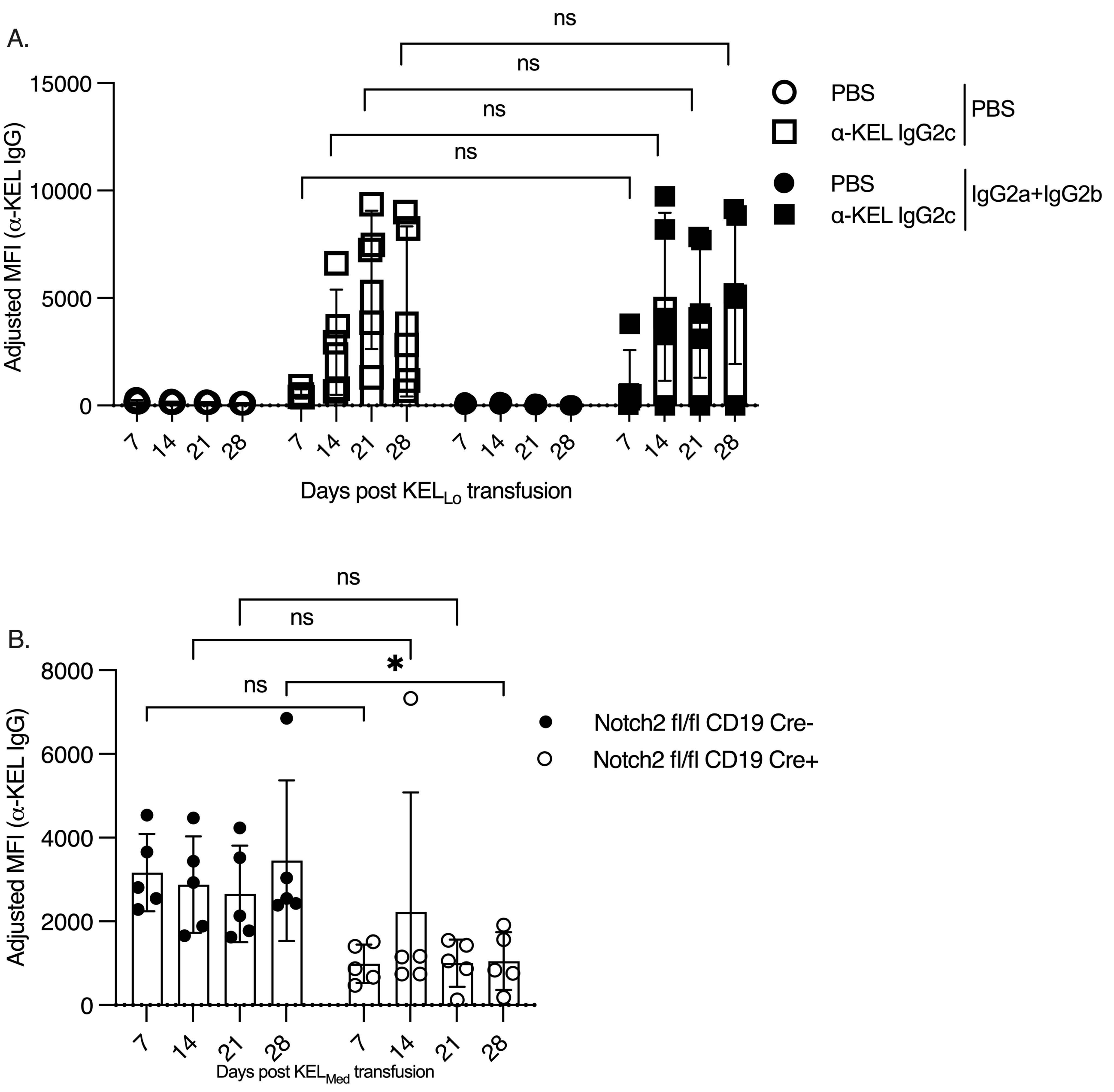

Supplemental Figure 4

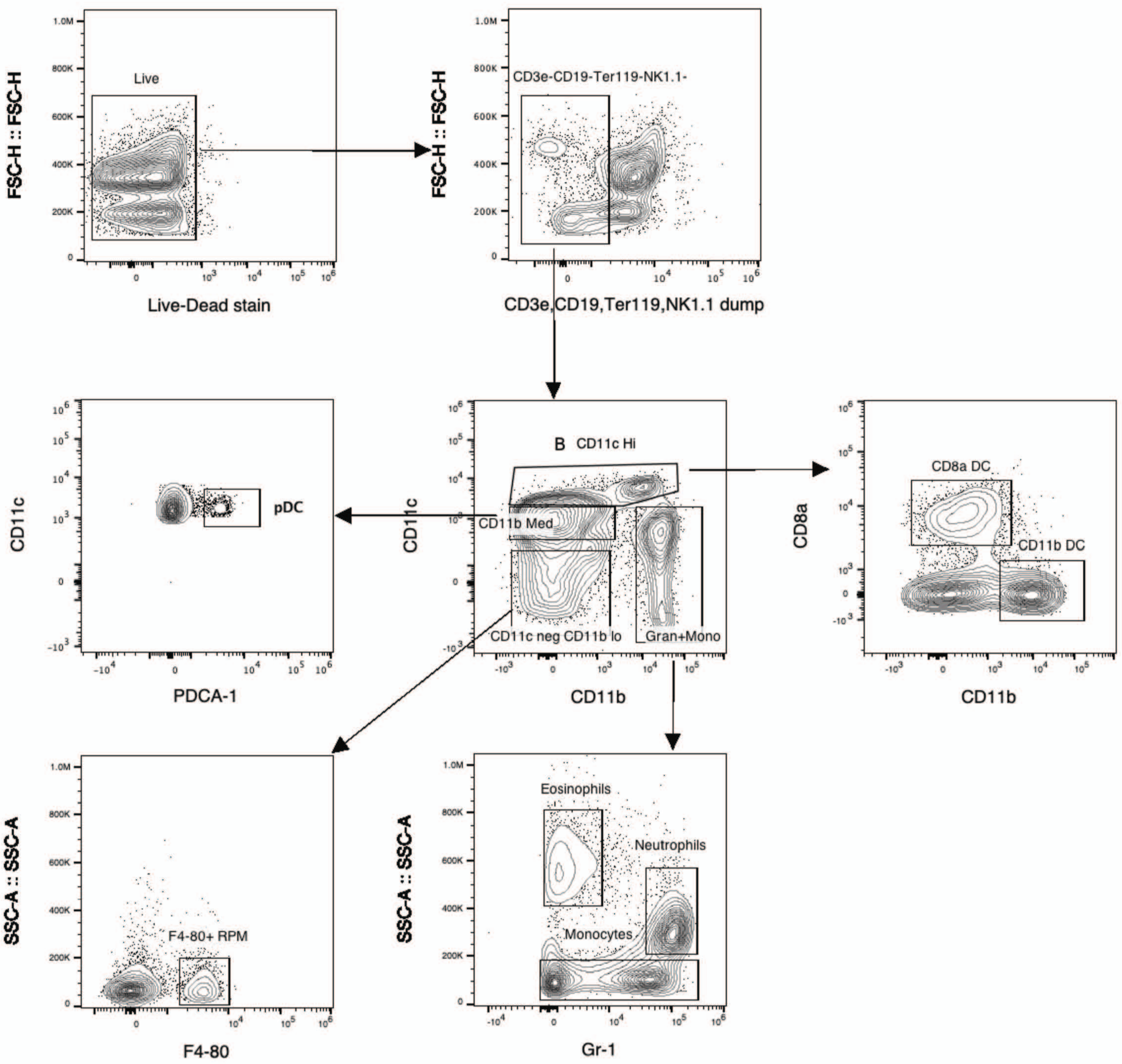

Supplemental Materials and Methods

Expression of soluble KEL (sKEL) and staining of B cells for sKEL reactivity:

The extracellular domain of the human Kel glycoprotein was cloned downstream of a 3X FLAG TAG with a single mutation of a cysteine residue precisely as previously described and without any deviation.(43) sKEL was expressed in transfected CHO cells and crude supernatants were used for staining of B cells. The presence of sKEL in the supernatants was confirmed both by Western Blot (using anti-FLAG) and by ELISA where anti-FLAG was coated on plates, sKEL was captured, and then PUMA4 was used as a detection reagent to ensure the sKEL had the correct conformation. Both Western Blot and ELISA confirmed the presence of sKEL (data not shown). Isolated splenocytes were incubated with sKEL containing supernatant, washed, and stained with PUMA3 conjugated to APC. PUMA3 has been previously described in detail. (37) In some cases, anti-FLAG APC was also used to control for the possibility that a significant number of B cells may recognize the same epitope as recognized by PUMA3. PUMA3 and anti-FLAG staining gave identical results.

Generation of Kp^bTg and Kp^bTg_{GL} mice:

The generation and characterization of anti-Kp^b (clone PUMA4), sequencing of the heavy and light chain, and expression of recombinant immunoglobulin have each been described in detail. (37) To generate BCR transgenic mice capable of normal class switching, the rearranged heavy chain and light chain sequences were targeted to the 5' end of the endogenous Ig_H or $I\kappa_H$ loci, respectively. This was carried out by the general strategy described by Taki et al.(77) and by the specific methods described by Phan et al. (78) Fine details of the generation and characterization are part of a manuscript currently in preparation and will be made available as part of the current manuscript, upon request. To include sufficient detail to allow reproduction of our experiments, the nucleic acid and protein sequences of PUMA4 are provided below.

To generate a low affinity PUMA4 variant, based upon the sequence, the original V, D, and J (for heavy chain) regions were inferred and DNA sequence was mutated to code for germ line V, D, and J sequences (prior to somatic hypermutation). The same approach was taken for the light chain sequence. Separate targeting constructs were generated for the germ line sequences and separate mice were generated. Founders with correct knockin of either heavy or light chain sequences were crossed to generate the final BCR mice.

<u>**PUMA4 sequences**</u> (for protein sequence, the <u>signal peptides are underlined.</u> Mutant amino acids in germ line sequences are indicated in bold.

Heavy Chain DNA:

ATGTACTTGGGACTGAACTGTGTATTCATAGTTTTTCTCTTAAAAGGTGTCCAGAGTGAAGT
GAAGCTTGAGGAGTCTGGAGGAGGCTTGGTGCAACCTGGAGGATCCATGAAACTCTCCTG
TGTTGCCTCTGGATTCACTTTCAGTAACTACTGGATGAACTGGGTCCGCCAACCTCCAGAG
AAGGGGCTTGAATGGGTTGCTGAAATTAGATTGAACTCTAATAATTATGCAACACATTATGC
GGAGTCTGTGAAAGGGAAATTCACCATCTCAAGAGATGATTCCAAAAGTAGTGTCTACCTG
CAAATGAACGACTTAAGAGCTGAAGACACTGGCATTTATTACTGTACCAGAAACTGGGACT
TTGCCTGGTTTGATTCCTGGGGCCAAGGGACTCTGGTCACTGTCTCTGCA

Heavy Chain Protein:

<u>MYLGLNCVFIVFLLKGVQS</u>EVKLEESGGGLVQPGGSMKLSCVASGFTFSNYWMNWVRQPPEK
GLEWVAEIRLNSNNYATHYAESVKGKFTISRDDSKSSVYLQMNDLRAEDTGIYYCTRNWDFAW
FDSWGQGTLVTVSA

Light Chain DNA:

ATGGAGTCACAGATTCAGGCATTTGTATTCGTGTTTCTCTGGTTGTCTGGTGTTGACGGAG
ACATTGTGATGACCCAGTCTCACAAATTCATGTCCACATCAGTAGGAGACAGGGTCAGCAT
CACCTGCAAGGCCAGTCAAGATGTGAGTACTGTTGTGGCCTGGTATCAACAAAAACCAGG
GCAATCTCCTAAACTACTGATTTACTGGGCATCCACCCGGCACACTGGAGTCCCTGATCGC
TTCACAGGCAGTGGATCTGGGACAGATTATACTCTCACCATCAGCAGTGTGCAGGCTGAA
GACCTGGCACTTTATTACTGTCAGCAACATTATACCACTCCATTCACGTTCGGCTCGGGGA
CAAAGTTGGAAATAAAA

Light Chain Protein:

MESQIQAFVFVFLWLSGVDGDIVMTQSHKFMSTSVGDRVSITCKASQDVSTVVAWYQQKPGQ
SPKLLIYWASTRHTGVPDRFTGSGSGTDYTLTISSVQAEDLALYYCQQHYTTPFTFGSGTKLEIK

PUMA4 sequences – Germ-line versions:

Heavy Chain DNA:

ATGTACTTGGGACTGAACTGTGTATTCATAGTTTTTCTCTTAAAAGGTGTCCAGAGTGAAGT
GAAGCTTGAGGAGTCTGGAGGAGGCTTGGTGCAACCTGGAGGATCCATGAAACTCTCCTG
TGTTGCCTCTGGATTCACTTTCAGTAACTACTGGATGAACTGGGTCCGCCAGTCTCCAGAG
AAGGGGCTTGAGTGGGTTGCTGAAATTAGATTGAAATCTAATAATTATGCAACACATTATGC
GGAGTCTGTGAAAGGGAGGTTCACCATCTCAAGAGATGATTCCAAAAGTAGTGTCTACCTG
CAAATGAACAACTTAAGAGCTGAAGACACTGGCATTTATTACTGTACCAGAAACTGGGACTT
TGCCTGGTTTGCTTACTGGGGCCAAGGGACTCTGGTCACTGTCTCTGCA

Heavy Chain Protein:

MYLGLNCVFIVFLLKGVQSEVKLEESGGGLVQPGGSMKLSCVASGFTFSNYWMNWVRQ**S**PEK
GLEWVAEIRL**K**SNNYATHYAESVKG**R**FTISRDDSKSSVYLQMN**N**LRAEDTGIYYCTRNWDFAW
F**AY**WGQGTLVTVSA

Light Chain DNA:

ATGGAGTCACAGATTCAGGCATTTGTATTCGTGTTTCTCTGGTTGTCTGGTGTTGACGGAG
ACATTGTGATGACCCAGTCTCACAAATTCATGTCCACATCAGTAGGAGACAGGGTCAGCAT
CACCTGCAAGGCCAGTCAGGATGTGAGTACTGCTGTAGCCTGGTATCAACAAAAACCAGG
GCAATCTCCTAAACTACTGATTTACTGGGCATCCACCCGGCACACTGGAGTCCCTGATCGC
TTCACAGGCAGTGGATCTGGGACAGATTATACTCTCACCATCAGCAGTGTGCAGGCTGAA
GACCTGGCACTTTATTACTGTCAGCAACATTATAGCACTCCATTCACGTTCGGCTCGGGGA
CAAAGTTGGAAATAAAA

Light Chain Protein:

MESQIQAFVFVFLWLSGVDGDIVMTQSHKFMSTSVGDRVSITCKASQDVST**A**VAWYQQKPGQ
SPKLLIYWASTRHTGVPDRFTGSGSGTDYTLTISSVQAEDLALYYCQQHY**S**TPFTFGSGTKLEI
K

Figure Legends

Figure 1 (Supplemental) Positive control for staining with anti-C3 antibody on RBCs. Red blood cells were incubated with rabbit anti-mouse RBC antisera and then incubated with plasma from either wild-type mice or C3 KO mice. After washing, treated RBCs were then stained with

anti-C3 as per main methods. As predicted, incubation with wild-type plasma (left panel) but not C3 KO plasma (middle panel) resulted in a 4 decade shift when staining with anti-C3. Histogram overlays from panels A and B are shown in panel C for direct comparison.

Figure 2 (Supplemental) Kinetics of phagocyte consumption of RBCs during AMIE. (A) The general experimental design (Figure 1 main manuscript) was modified by using RBCs that were from an F1 cross from KEL-$K2_{Lo}$ mice and GFP transgenic mice. Dual expression of KEL-$K2_{Lo}$ and GFP were confirmed by flow cytometry by staining with anti-Kp^b. As predicted, only KEL-$K2_{Lo}$.GFP RBCs were positive for both anti-Kp^b and GFP. No decrease in staining for KEL-$K2_{Lo}$ or GFP was detected in the KEL-$K2_{Lo}$.GFP RBCs compared to the single transgenic strains. **(B)** The acquisition of GFP fluorescence in each cell population (as per main paper) was analyzed at 15 min, 1 hr, 6 hrs, and 24 hrs post transfusion in the absence or presence of anti-Kp^b IgG2c. A significant increase was seen in anti-Kp^b treated mice at 1 and 6 hours post transfusion. **(C)** Representative flow cytometry plots are shown to show the magnitude of GFP acquisition by RPM. **(D)** Additional mice from the same experiment were allowed to proceed to 21 days post-transfusion and plasma was tested for anti-KEL in order to ensure that the addition of GFP into the system did not alter IgG2c induced AMIE phenomenon. IgG2c induced AMIE was unaffected by GFP. P values were calculated using a repeated measures two-way ANOVA with Sidak multiple comparison test and are designated as (nonsignificant (ns)= >0.05), (*=<0.05), (**=<0.01), (***=<0.001).

Figure 3 (Supplemental) Controls for nonspecific effects of isotype controls and for correct phenotype of conditional CD19 Notch2 fl/fl KO mice. (A) To test if the injection of isotype matched controls for the MZ depletion protocol had any non-specific effects of immunoglobulin injection, groups injected with isotype matched controls or PBS were compared. IgG2c induced AMIE was unaltered by the injection of isotype matched controls (IgG2a + IgG2b). **(B)** It has been previously reported that CD19CRE x Notch2 fl/fl mice have

decreased antibody responses to transfusion of KEL-K2$_{Med}$ RBCs. The mice generated for the current studies had the same blunted immune response as previously reported, confirming the phenotype of the mice. P values were calculated using a repeated measures two-way ANOVA with Sidak multiple comparison test and are designated as (nonsignificant (ns)= >0.05), (*=<0.05), (**=<0.01).

Figure 4 (Supplemental) Gating strategy for distinguishing different potentially phagocytic populations to determine consumption of GFP+ RBCs during AMIE. An example flow cytometry play is shown with each population indicated. The panel of antibodies used is described in the main materials and methods.
